# Vaccine Hesitancy Among Parents in Croatia: Findings from a Nationwide PACV-Based Cross-Sectional Study

**DOI:** 10.3390/children13030403

**Published:** 2026-03-14

**Authors:** Lidija Tubikanec, Ana Marija Švigir, Martina Smrekar, Snježana Čukljek, Sanja Ledinski, Boris Ilić, Biljana Filipović

**Affiliations:** 1 Health Care Center Zagreb “Centar”, 10000 Zagreb, Croatia; lidija.tubikanec@dzz-centar.hr; 2 Department of Nursing, University of Applied Health Sciences, 10000 Zagreb, Croatia; martina.smrekar@zvu.hr (M.S.); snjezana.cukljek@zvu.hr (S.Č.); sanja.ledinski@zvu.hr (S.L.); boris.ilic@zvu.hr (B.I.); biljana.filipovic@zvu.hr (B.F.)

**Keywords:** vaccine hesitancy, PACV, parents, childhood vaccination, Croatia, pediatric nursing

## Abstract

**Highlights:**

**What are the main findings?**

- A substantial proportion of Croatian parents demonstrate childhood vaccine hesitancy.
- Higher parental education was associated with lower PACV scores

**What are the implications of the main findings?**

- Addressing parental concerns through structured communication may improve vaccine confidence.
- Pediatricians and community health nurses play a key role in supporting informed vaccination decisions.

**Abstract:**

**Background**: Childhood vaccination remains one of the most effective public health interventions. Despite consistently high national coverage, vaccine hesitancy persists among parents and may undermine herd immunity. The Parent Attitudes about Childhood Vaccines (PACV) questionnaire provides a validated framework for identifying parental concerns and patterns of hesitancy. **Methods**: A cross-sectional online survey was conducted in May 2025 using the Croatian version of the Parent Attitudes about Childhood Vaccines (PACV) questionnaire. The study included 1087 parents aged 18–65 years. PACV scores were transformed to a 0–100 scale, with values ≥50 indicating vaccine hesitancy. Associations between PACV scores and parental age and educational level were analysed using non-parametric statistical tests. **Results**: Most respondents were mothers (87.7%) and aged between 30 and 45 years (71.8%). Approximately one fifth of parents reported postponing vaccination (22.7%), and 19.2% indicated having refused at least one vaccine dose. While 63.7% expressed full acceptance of recommended childhood vaccines, a substantial proportion either refused vaccination (20.8%) or remained undecided (15.5%). Higher educational attainment was significantly associated with lower PACV scores, whereas no significant association was observed with parental age. **Conclusions**: Although overall vaccination acceptance in Croatia remains high, vaccine hesitancy continues to affect a considerable proportion of parents. Strengthening tailored communication strategies and reinforcing trust-based counselling—particularly within pediatric and community nursing services—may support informed decision-making and improve vaccine confidence.

## 1. Introduction

Childhood vaccination represents one of the most effective and cost-efficient public health interventions for preventing infectious diseases and reducing childhood morbidity and mortality worldwide. Maintaining high vaccination coverage is essential for preserving herd immunity and protecting vulnerable populations who cannot be vaccinated due to medical contraindications. Despite the well-established safety and effectiveness of vaccines, parental vaccine hesitancy remains a growing global public health challenge and has been identified by the World Health Organization as a major threat to global health. Multiple factors contribute to vaccine hesitancy, including misinformation, cultural beliefs, and distrust in healthcare systems [1,2,3,4].

In recent years, the rapid dissemination of health-related content through social media has further amplified parental uncertainty regarding childhood immunization. Online platforms often facilitate the spread of misinformation, which can negatively influence perceptions of vaccine safety and efficacy [5,6,7]. Exposure to inaccurate or conflicting information has been associated with increased parental anxiety about potential adverse effects and reduced vaccine uptake [5,8]. The COVID-19 pandemic further demonstrated how digital environments can shape vaccination attitudes, even among populations previously compliant with immunization recommendations [5,9].

Vaccine hesitancy is defined as the delay in acceptance or refusal of vaccination despite the availability of vaccination services and reflects a complex interaction between cognitive, emotional, and contextual factors influencing parental decision-making [5,6,10]. Research indicates that parental experiences with healthcare providers and the credibility of information sources significantly influence hesitancy levels [11,12,13].

Croatia maintains a long-standing mandatory childhood immunization program with relatively high overall vaccination coverage. However, emerging evidence suggests that an increasing proportion of parents express doubts, postpone vaccinations, or selectively refuse certain vaccines. This trend poses potential risks for the re-emergence of vaccine-preventable diseases and highlights the importance of continuous monitoring of parental attitudes toward vaccination [14,15].

The Parent Attitudes about Childhood Vaccines (PACV) questionnaire was developed as a standardized tool to assess parental beliefs, behaviors, and concerns related to childhood vaccination [15,16,17]. The instrument has demonstrated strong predictive validity, with higher hesitancy scores associated with incomplete childhood immunization status [16]. Furthermore, the questionnaire has been successfully translated and validated across multiple countries, including Croatia, where the Croatian version has shown high internal consistency and satisfactory psychometric properties [14].

Beyond its role as a research instrument, the PACV questionnaire also offers practical value for healthcare professionals by enabling early identification of hesitant parents and facilitating targeted communication strategies. Pediatric healthcare providers and community nursing services play a crucial role in addressing parental concerns, building trust, and supporting informed decision-making regarding childhood vaccination [15]. Recent Croatian studies have examined parental vaccine hesitancy using standardized instruments, including large-scale online surveys. These studies have provided important baseline insights into the prevalence and structure of vaccine hesitancy in Croatia. However, given the dynamic and context-dependent nature of vaccine attitudes, continued research across independent samples remains essential to monitor trends, explore sociodemographic correlates, and ensure stability of findings. Replication using comparable validated instruments contributes to the robustness and cumulative strength of national evidence [14].

Understanding these patterns is essential for designing evidence-based interventions and strengthening vaccination promotion strategies within pediatric and community healthcare settings.

Vaccine hesitancy among parents in Croatia can be further understood within the WHO “3C” framework, which conceptualizes hesitancy through confidence, convenience, and complacency. Confidence refers to trust in vaccine safety, effectiveness, and healthcare providers; convenience encompasses communication channels, service accessibility, and logistical barriers; and complacency reflects parents’ perception of disease risk and the perceived necessity of vaccination. Interpreting PACV findings within this framework may support the identification of dominant determinants of hesitancy and inform targeted public health strategies [18].

In Croatia, childhood vaccination coverage has traditionally been high due to a long-standing mandatory immunization program [19]. However, recent years have shown fluctuations in vaccination confidence and periodic declines in coverage for certain vaccines, particularly measles-containing vaccines. Similar patterns have been associated with localized measles clusters and public health alerts in Croatia and other European countries [20]. These developments emphasize the need for continued monitoring of parental vaccine attitudes.

This study aimed to examine parental attitudes toward childhood vaccination in Croatia using the PACV questionnaire and to explore the association between vaccine hesitancy and parental age and educational level in order to support evidence-based pediatric and community healthcare practice.

Based on previous literature and the study objectives, we formulated the following hypotheses:

**H1.**  *Parental vaccine hesitancy differs according to educational level.*

**H2.**  *Parental vaccine hesitancy differs according to parental age.*

## 2. Materials and Methods

### 2.1. Study Design

A cross-sectional quantitative study was conducted to assess parental attitudes toward childhood vaccination using the validated Parent Attitudes about Childhood Vaccines (PACV) questionnaire. Data collection was performed in May 2025 using an anonymous online survey format.

### 2.2. Participants and Recruitment

The study included parents aged between 18 and 65 years who had at least one child and completed all PACV questionnaire items. Participation was voluntary and anonymous. The survey link was distributed via social media platforms, enabling access to a geographically diverse sample across Croatia. Incomplete questionnaires were automatically excluded by the survey platform settings.

A total of 1087 respondents met the inclusion criteria and were included in the final analysis.

Given the large sample size (n = 1087), the study provides a robust descriptive overview of parental attitudes toward childhood vaccination in Croatia. The use of a validated and widely applied instrument (PACV) further strengthens the methodological reliability and comparability of findings with international research. Although the study was not designed to test causal relationships, the sample size allows for stable estimation of patterns of vaccine hesitancy and meaningful subgroup comparisons.

Participants were recruited via a snowball sampling strategy through multiple online channels, including Facebook parenting groups, WhatsApp parent networks, and mailing lists distributed through primary healthcare settings. No financial or material incentives were provided. Due to the open online distribution method, complete technical control over duplicate submissions was not possible. Technical restrictions such as IP tracking were not implemented in order to preserve anonymity; however, no irregular response patterns suggesting duplication were detected during data screening.

### 2.3. Instrument

Parental attitudes toward childhood vaccination were assessed using the Parent Attitudes about Childhood Vaccines (PACV) questionnaire. The Croatian validated version of the instrument was used with formal permission from the original questionnaire author (Douglas Opel, University of Washington) and the Croatian adaptation authors (Ćurkovit and Matana, University of Split, Croatia). The use of a previously validated Croatian version ensured methodological consistency, cultural appropriateness, and comparability with national and international studies on vaccine hesitancy.

The PACV questionnaire consists of 15 items covering three domains:

1. Vaccination-related behaviors
2. Perceived vaccine safety and effectiveness
3. General trust in vaccines and the healthcare system

Response formats include dichotomous items (Yes/No/Unsure), Likert-type scales, and numerical rating scales. Responses were recoded into three categories (0 = non-hesitant, 1 = unsure, 2 = hesitant) according to the original PACV scoring protocol before calculating the total score. Item scoring follows the original PACV scoring protocol. Raw scores are summed and linearly transformed to a 0–100 scale, where higher scores indicate greater vaccine hesitancy. A score ≥ 50 is commonly used as the cutoff value indicating the presence of vaccine hesitancy.

The internal consistency of the PACV version in the present sample was acceptable, with a Cronbach’s alpha of 0.922 (N = 1086).

### 2.4. Sociodemographic Variables

In addition to PACV items, the survey collected sociodemographic data including:

- gender
- age
- educational level
- marital status
- number of children
- county of residence

Age was categorized into three groups: 18–29 years, 30–45 years, and 46–65 years. Educational level was classified as primary education, secondary education, university degree, and postgraduate education.

### 2.5. Ethical Considerations

The study was conducted in accordance with the principles of the Declaration of Helsinki. Participation was voluntary and anonymous, and informed consent was implied through completion of the online questionnaire. All participants were informed about the study’s purpose and data confidentiality prior to participation.

Ethical approval was obtained from the Ethics Committee of the University of Applied Health Sciences Zagreb (Approval number: 053-01/25-01/04; code: 251-379-10-25-02).

### 2.6. Statistical Analysis

Normality of the PACV score distribution within age and education subgroups was assessed using the Kolmogorov–Smirnov test (Lilliefors correction) and the Shapiro–Wilk test. Because the PACV score distributions deviated from normality in most subgroups, non-parametric methods were applied. Although several sociodemographic variables were collected to describe the study population, inferential analyses focused on parental age and educational level, in line with the predefined study objectives and hypotheses and previous literature identifying these variables as key determinants of vaccine hesitancy. Given the exploratory cross-sectional design and predefined study hypotheses, analyses focused on bivariate comparisons between PACV scores and key sociodemographic variables. Differences in PACV scores across parental age groups (18–29, 30–45, 46–65 years) and education levels (primary, secondary, university, postgraduate) were examined using the Kruskal–Wallis test. When the Kruskal–Wallis test indicated statistically significant differences, post hoc pairwise comparisons were performed with Bonferroni-adjusted significance values. A two-sided *p*-value < 0.05 was considered statistically significant. An a priori power analysis was not performed due to the exploratory cross-sectional design and the snowball recruitment approach, which aimed to obtain the largest feasible sample. Effect sizes are reported to support interpretation of the magnitude of observed differences.

PACV scores were analyzed both on the original 0–30 scale and on the transformed 0–100 scale according to the original PACV scoring protocol.

## 3. Results

### 3.1. Sample Characteristics

Overall, the findings indicate that vaccine hesitancy is present in a substantial proportion of Croatian parents.

The final sample comprised 1087 parents, the majority of whom were mothers (87.7%). Most participants were aged 30–45 years (71.8%). More than half had completed university education (53.1%), while 12.2% reported postgraduate qualifications. Most respondents were married or living in a partnership (91.7%), and nearly half reported having two children (45.1%).

PACV scores showed a moderately right-skewed distribution (skewness = 0.461).

The overall PACV score had a median value of 10 (IQR = 14) on the original 0–30 scale, corresponding to 33.3 on the transformed 0–100 scale. After linear transformation to the 0–100 scale, the mean score was 38.8 ± 29.1. Using the established cutoff value (≥50), 36.9% of parents were classified as vaccine-hesitant (Table 1).

**Table 1:** PACV hesitant.

	Frequency	Percent
Non-hesitant	686	63.1
Hesitant	401	36.9
Total	1087	100.0

### 3.2. Vaccination Behaviour and Intentions

With regard to vaccination behaviour, 22.7% of parents indicated that they had postponed at least one childhood vaccination for reasons unrelated to illness or allergy. In addition, 19.2% reported having refused at least one vaccine dose. When asked about future vaccination intentions, 63.7% stated that they would accept all recommended vaccines. However, 20.8% reported unwillingness to vaccinate and 15.5% remained uncertain, reflecting ongoing hesitation among a substantial subgroup of parents.

### 3.3. Sociodemographic Associations with PACV Scores

Parental age was not significantly associated with PACV scores (H(2) = 2.437, *p* = 0.296, η^2^ = 0.0004), indicating a negligible effect size. Median PACV scores were comparable across age groups.

In contrast, educational level was significantly associated with PACV scores (H(3) = 16.71, *p* < 0.001, η^2^ = 0.013), indicating a small effect size. Median PACV scores decreased with higher educational attainment, from 40.00 among parents with primary and secondary education to 33.33 among those with a university degree and 23.33 among parents with postgraduate education (Table 2). Post-hoc pairwise comparisons with Bonferroni correction showed that parents with secondary education had significantly higher PACV scores than those with university (*p* = 0.008) and postgraduate education (*p* = 0.002), while other comparisons were not statistically significant.

## 4. Discussion

The present study provides one of the largest contemporary datasets on parental vaccine attitudes in Croatia. The substantial sample size and use of a validated measurement tool enhance the reliability of the findings and enable meaningful comparison with international studies addressing vaccine hesitancy. The relatively high proportion of hesitant parents observed in this online sample may partly reflect the recruitment method, as individuals with stronger opinions about vaccination may be more likely to participate in voluntary online surveys. This subgroup of hesitant but compliant parents represents an important target for communication strategies within pediatric and community nursing practice. Although 36.9% of parents were classified as vaccine-hesitant according to the PACV score, a smaller proportion reported actual refusal of vaccination. This suggests that a substantial group of hesitant parents still accepts vaccination, highlighting an important target population for communication-based interventions. This distinction highlights the importance of differentiating between vaccine hesitancy and vaccine refusal, as hesitant parents may still be receptive to targeted communication and supportive counselling from healthcare professionals.

This study offers large-scale national evidence on parental attitudes toward childhood vaccination in Croatia and highlights the continued presence of vaccine hesitancy despite long-standing mandatory immunisation programmes. Although overall vaccination coverage in Croatia remains relatively high compared with many European countries, pockets of hesitancy may still pose risks for local outbreaks of vaccine-preventable diseases. One of the most important findings was the association between educational level and vaccine hesitancy. Parents with higher educational attainment demonstrated lower PACV scores. Our findings are consistent with previous studies showing that higher parental education is associated with greater acceptance of childhood vaccination. Associations between educational level and vaccine hesitancy vary across studies and contexts. Rather than representing a simple knowledge deficit, this pattern appears to be linked to more complex processes involving risk perception, information overload, and trust in public health authorities [21,22,23].

Post-hoc comparisons further indicated that the observed difference was primarily driven by higher hesitancy among parents with secondary education compared with those holding university or postgraduate degrees, suggesting that tertiary education may play a role in strengthening vaccine confidence.

Although previous research has sometimes linked higher education with increased critical appraisal of health information, our findings suggest that in this sample higher educational attainment may be associated with greater confidence in vaccination. This aligns with findings from Derdemezis et al., who examined vaccine hesitancy in Greek parents, asserting that educational background plays a significant role in shaping vaccine attitudes [24]. Similarly, studies conducted in Ireland using the PACV highlighted that the complexities of vaccine hesitancy are not merely about knowledge but also interlink deeply with personal beliefs and experiences related to healthcare systems [22].

In contrast, parental age was not associated with PACV scores. This finding suggests that vaccine hesitancy is not confined to a particular age cohort and may instead be shaped by broader psychosocial and contextual factors. Trust in healthcare professionals, perceived vaccine safety, and previous experiences with the healthcare system may play a more central role than chronological age alone [22,23,25].

Understanding these dynamics is crucial for developing effective public health interventions. A systematic review by Dyda et al. highlights the importance of addressing parental attitudes and beliefs as these are key factors influencing vaccine acceptance [23]. Moreover, qualitative findings indicate that parents are often navigating a landscape filled with misinformation, which can lead to heightened concern [22,24]. Consequently, healthcare providers may need to approach parental concerns in a nuanced manner, recognizing that the educational background can shape how parents interpret vaccine-related information.

From a clinical perspective, these findings emphasise the importance of proactive engagement within pediatric primary care. Pediatricians and community health nurses are often the first point of contact for parents and are therefore well positioned to identify early signs of hesitancy. Although the PACV has demonstrated predictive validity in previous research, the present study did not assess behavioral vaccination outcomes; therefore, its role as a clinical screening instrument should be interpreted with caution.

Our findings also illustrate the important distinction between vaccine hesitancy and vaccine refusal. Although a substantial proportion of parents were classified as hesitant according to the PACV scoring system, the majority still reported accepting recommended childhood vaccinations.

Structured communication approaches, including motivational interviewing and shared decision-making, may further enhance parental engagement and trust. Community health nurses, in particular, play a key role in maintaining continuity of care and reinforcing evidence-based vaccination messages in a supportive and non-confrontational manner. Strengthening interdisciplinary collaboration between pediatricians, nurses, and public health institutions may further improve the consistency and effectiveness of vaccination promotion strategies.

Several limitations should be acknowledged. First, the study was based on an online snowball sampling approach, which does not allow full representativeness of the Croatian parent population. Participation was voluntary and dependent on digital access and social network dissemination, which may have introduced selection bias and resulted in overrepresentation of more digitally active or health-engaged parents. Consequently, the findings may not fully reflect the attitudes of parents with limited internet access or lower online engagement. Second, the predominance of mothers (87.7%) limits the generalisability of the findings to fathers and may reflect gender differences in engagement with child health topics. Third, although measures were taken to ensure anonymity, the open online distribution method did not allow complete technical control over potential duplicate responses. Finally, due to the cross-sectional design, causal relationships between sociodemographic characteristics and vaccine hesitancy cannot be established. An important observation in this study is that the proportion of parents classified as vaccine-hesitant (36.9%) was considerably higher than the proportion reporting refusal of childhood vaccines. This finding supports the conceptual distinction between vaccine hesitancy and vaccine refusal, indicating that many parents who express concerns about vaccination still ultimately accept recommended immunizations.

**Implications** **for Pediatric and Nursing Practice**

The findings of this study have several important implications for pediatric and community healthcare practice. Routine assessment of parental vaccine attitudes using validated tools such as the PACV questionnaire may support the identification of parental concerns; however, further research linking PACV scores with actual vaccination behavior is warranted before firm clinical screening recommendations can be made.

Community health nurses are uniquely positioned to support vaccination programs through direct contact with families during home visits, well-child appointments, and preventive health consultations. Their role in building long-term trust relationships with parents provides an opportunity to address concerns, clarify misconceptions, and reinforce evidence-based vaccination messages.

Implementing structured communication strategies, including motivational interviewing and shared decision-making approaches, may improve parental engagement and reduce resistance to vaccination. Healthcare professionals should be trained to recognize different types of hesitancy and adapt their communication style accordingly, focusing on empathy, transparency, and respectful dialogue.

Collaboration between pediatricians, nurses, and public health institutions should be strengthened to ensure consistent messaging and coordinated vaccination promotion strategies. Developing targeted educational materials for highly educated parents—who may actively seek information from diverse sources—could further improve the effectiveness of vaccination campaigns.

Integrating vaccination attitude assessment into routine pediatric care and enhancing interdisciplinary collaboration may contribute to improved vaccine confidence and sustained childhood immunization coverage.

## 5. Conclusions

This study demonstrates that, despite high overall childhood vaccination coverage in Croatia, vaccine hesitancy remains a relevant public health concern. A substantial proportion of parents report postponing, refusing, or expressing uncertainty about recommended childhood vaccines. The observed association between higher educational attainment and lower PACV scores highlights the complexity of contemporary vaccine decision-making. The absence of an age-related effect suggests that vaccine hesitancy affects parents across different life stages and should be addressed broadly within pediatric healthcare services. Strengthening trust-based communication, improving personalised counselling strategies, and integrating structured assessment tools into routine care may contribute to improved vaccine confidence and sustained immunisation uptake. Future research should focus on longitudinal trends and intervention-based approaches aimed at reducing parental hesitancy and supporting informed vaccination decisions.

## Figures and Tables

**Table 2:** PACV scores according to parental education level.

Education Level	N	Median (IQR)
Primary education	9	40.00 (51.67)
Secondary education	367	40.00 (46.67)
University degree	577	33.33 (50.00)
Postgraduate education	133	23.33 (43.33)

Kruskal–Wallis test: H(3) = 16.71, *p* < 0.001. Effect size: η^2^ = 0.013 (small effect).

## Data Availability

The data supporting the findings of this study are available from the corresponding author upon reasonable request. The data are not publicly available due to privacy and ethical restrictions related to the survey participants.
